# Neutral Polymorphisms in Putative Housekeeping Genes and Tandem Repeats Unravels the Population Genetics and Evolutionary History of *Plasmodium vivax* in India

**DOI:** 10.1371/journal.pntd.0002425

**Published:** 2013-09-19

**Authors:** Surendra K. Prajapati, Hema Joshi, Jane M. Carlton, M. Alam Rizvi

**Affiliations:** 1 Molecular Biology Division, National Institute of Malaria Research, New Delhi, India; 2 Center for Genomics and System Biology, Department of Biology, New York University, New York, New York, United States of America; 3 Department of Biosciences, Jamia Millia Islamia University, New Delhi, India; Barcelona Centre for International Health Research (CRESIB) and Institució Catalana de Recerca i Estudis Avançats (ICREA), Spain

## Abstract

The evolutionary history and age of *Plasmodium vivax* has been inferred as both recent and ancient by several studies, mainly using mitochondrial genome diversity. Here we address the age of *P. vivax* on the Indian subcontinent using selectively neutral housekeeping genes and tandem repeat loci. Analysis of ten housekeeping genes revealed a substantial number of SNPs (n = 75) from 100 *P. vivax* isolates collected from five geographical regions of India. Neutrality tests showed a majority of the housekeeping genes were selectively neutral, confirming the suitability of housekeeping genes for inferring the evolutionary history of *P. vivax*. In addition, a genetic differentiation test using housekeeping gene polymorphism data showed a lack of geographical structuring between the five regions of India. The coalescence analysis of the time to the most recent common ancestor estimate yielded an ancient TMRCA (232,228 to 303,030 years) and long-term population history (79,235 to 104,008) of extant *P. vivax* on the Indian subcontinent. Analysis of 18 tandem repeat loci polymorphisms showed substantial allelic diversity and heterozygosity per locus, and analysis of potential bottlenecks revealed the signature of a stable *P. vivax* population, further corroborating our ancient age estimates. For the first time we report a comparable evolutionary history of *P. vivax* inferred by nuclear genetic markers (putative housekeeping genes) to that inferred from mitochondrial genome diversity.

## Author Summary

*Plasmodium vivax* is the most prevalent human malaria parasite outside Africa, responsible for significant morbidity, although it is commonly referred to as a benign malaria agent with respect to *P. falciparum*. Inferring the evolutionary history of an infectious agent provides important information for designing control measures. In this study, we developed a panel of novel molecular markers including housekeeping genes, minisatellites and microsatellites to infer the evolutionary history of *P. vivax* on the Indian subcontinent. The panel of markers uncovered an ancient and long evolutionary history and a stable population structure of *P. vivax* in India.

## Introduction

*Plasmodium vivax* is the most prevalent malaria species outside Africa, causing widespread morbidity that can be severe and fatal [1], [2], [3], [4], [5], [6]. The fixation of the Duffy negativity trait (*P. vivax* resistance factor) in tropical African populations restricts infection by this parasite there [7]. *P. vivax* is distantly related to the more virulent *Plasmodium falciparum*, as inferred from mitochondrial and nuclear DNA variation [8], [9], [10], [11]; the former appears to have evolved from a monkey malaria parasite between 20–35 million years ago via host switching [12]. Various studies on the age and origin of extant *P. vivax* strongly support it as an ancient human malaria parasite that evolved in Southeast Asia [12], [13], [14], [15].

Single nucleotide polymorphisms (SNPs) have been used by several researchers to elucidate the population history of *P. falciparum* and *P. vivax* [12], [16], [17], [18]. These studies established the importance of using selectively neutral loci for determining the molecular age, origin, and evolutionary history of the parasites, assuming a “molecular clock” hypothesis. Many population studies have exploited polymorphisms in two kinds of neutral loci: SNPs in putative housekeeping genes, and repeat polymorphisms in tandem repeats such as microsatellites [19], [20], [21]. Genome sequencing of *P. vivax* has revealed a higher SNP density [22] and fewer microsatellite markers than in *P. falciparum* [23], [24].

India contributes about 78% of the total malaria cases in South Asia, and *P. vivax* accounts for 50–55% of this [25]. The paucity of information about the age and evolutionary history of *P. vivax* in Indian populations [15], [26] makes such investigations highly relevant. In this paper, we use an evolutionary genetics approach to unravel the evolutionary history of *P. vivax* on the Indian subcontinent. We have developed putative housekeeping gene, microsatellite, and minisatellite markers for investigating the population and evolutionary history of *P. vivax* on the Indian subcontinent.

## Materials and Methods

### Identification of putative housekeeping genes

**Figure 1:**
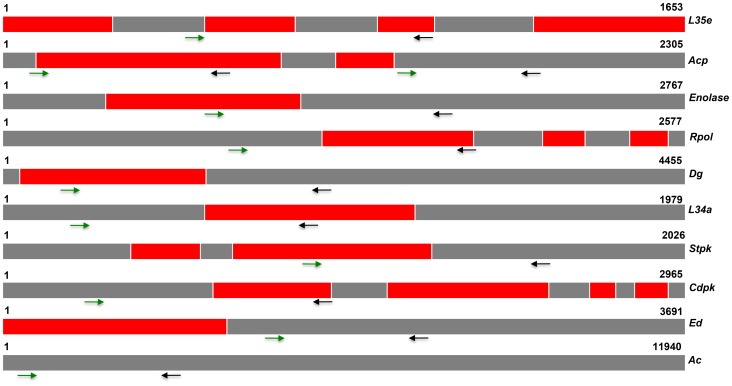
Schematic representation of *Plasmodium vivax* housekeeping genes and location of primers. Red box, exon; gray box, intron; green arrow, forward primer; black arrow, reverse primer; number indicates start and end of respective gene. Abbreviations: *L35e, Ribosomal protein l35e; ACP, Acyl carrier protein; Rpol, RNA polymerase-II; Dg, DNA gyrase; L34a, Ribosomal protein l34a; Stpk, Serine threonine protein kinase; Cdpk, Calcium dependent protein kinase; Ed, Exonuclease domain* and *Ac, Adenylate cyclase*.

Putative housekeeping genes were identified from the *P. vivax* genome database PlasmoDB (http://www.plasmodb.org). The major parameters used for selecting housekeeping genes from PlasmoDB were constitutive expression of gene, presence of orthologs in other malaria parasites, and an assigned metabolic function. For the detection of SNPs in putative housekeeping genes, both exons and introns from each gene were selected. PCR primers were manually designed and the location of each primer is shown in **Figure 1**.

### *Plasmodium vivax* isolates and DNA extraction

We analyzed 100 *P. vivax* field isolates collected between 2003–2006 from Delhi, Nadiad (Gujarat), Panna (Madhya Pradesh), Chennai (Tamil Nadu), and Kamrup (Assam), a total of twenty samples from each site (Figure S1). Human populations at these study sites do not have the Duffy negative trait [27]. Other details about the study sites such as parasite and vector species prevalence and disease transmission patterns are given in the Text S1 and reported elsewhere [28], [29]. DNA extraction was as described in reference [28], [29].

### Ethics statement

The ethics committee of the National Institute of Malaria Research approved the study protocol. All subjects provided informed consent, with children providing consent via a parent or guardian.

### PCR amplification, sequencing, and sequence analysis

PCR amplification reactions were carried out in a final volume of 20.0 µl that included 1–2 µl (∼3–5 ng) template DNA, 10 pM for each primer, and 2× Master Mix (Promega). PCR primers and the protocols used for amplification of the housekeeping genes are given in **Table S1**. PCR products were purified and sequenced commercially (Macrogen Inc, Seoul, Korea, http://dna.macrogen.com). For each gene, both forward and reverse primers were used in DNA sequencing. DNA Lasergene software (DNA Star Inc., USA) was used for editing raw DNA sequences (EditSeq Module), and sequences alignment (Clustal W module). Each mutation observed in a sample was confirmed by both forward and reverse sequences.

### Single clone infection typing

As multi-clone isolates could hamper correct genotyping and lead to over-estimating genetic diversity in a multi-locus genotyping study, *Pvmsp-3α* PCR-RFLP analysis was used to identify single- and multi-clone infections [30]; only single clone samples (n = 100) were used for genotyping.

### Tandem repeat genotyping

Tandem Repeat Finder version 4.00 [31] was used to identify microsatellites from the *P. vivax* genome. The characteristics of microsatellites are listed in **Table S2**. Each forward primer was modified with fluorescence dye, 6FAM, TET and HEX for accurate microsatellite allele sizing. Amplified PCR products were multiplexed with two different dyes and run on a DNA sequencer (ABI 3730: Applied Biosystems Inc. USA). Peak Scanner (Applied Biosystems, Foster City, CA) was used for microsatellite allele sizing.

### Measures of genetic diversity and neutrality

Genetic diversity was measured in the *P. vivax* population using parameters including nucleotide diversity (π), haplotype diversity (Hd) and average number of nucleotide differences (K) using DnaSP version 4.10 [32]. Microsatellite genetic diversity was measured by calculating expected heterozygosity (*He*) for each locus using MicroSatellite Analyzer (MSA) version 4.00 [33]. Expected heterozygosity (*He*) was defined as [n/(n−1)] [1−Σp_i_^2^], where n is the number of isolates analyzed and pi is the frequency of the i^th^ allele in the population.

### Genetic differentiation test

We estimated genetic differentiation between five geographical populations of *P. vivax* in the Indian subcontinent using DnaSP version 4.10 [32]. This software calculated chi-square (χ^2^) statistics of housekeeping gene haplotype frequencies between populations [34]. The significant *P*-value (<0.05) of χ^2^ statistics rejects null hypothesis. The null hypothesis assumes that all populations are genetically undifferentiated.

### Test of neutrality

We calculated D statistics [35], [36] and ratio of d_N_/d_S_ to detect signature of selection at putative housekeeping genes using DnaSP version 4.10 [32]. The number of segregating sites was used for inferring D test statistics. To establish the nature of selection at codons of putative housekeeping genes, we assessed the ratio of the rates of non-synonymous and synonymous substitutions.

### Estimation of divergence, TMRCA, and effective population size

The divergence time of human and monkey has been deduced as 30–35 million years ago (mya) [37], [38] which is consistent with 11–41 mya divergence time between *P. vivax* and nonhuman primate malarias [8]. We presumed a 23–30 mya divergence time between *P. vivax* and *P. knowlesi* that is coincident with their host divergence time [37], [38]; this divergence time was used in the estimation of mutation rate and synonymous divergence rate by comparing orthologous putative housekeeping genes of *P. vivax* and *P. knowlesi*. In addition, the recent divergence estimates between *P. vivax* and *P. knowlesi* (2 to 3 mya and 3.8 to 6.3 mya) was assumed on the basis of radiation of Asian macaques [13], [39]. These divergence estimates were also used for the calculation of the above molecular rates.

We estimated the Time to Most Recent Common Ancestor (TMRCA) and effective population size using SNPs present in putatively selectively neutral housekeeping genes. The TMRCA of *P. vivax* was determined by the equation d_S_ = 2 **μ**_s_×T*w*, where d_S_ is synonymous substitution rate at synonymous site, **μ**_s_ is synonymous mutation rate per site per year and T*w* is TMRCA [18]. Synonymous mutation rate was determined by the equation Ks = 2 **μ**_s_×T*b*+d_S_ [18], where Ks is the divergence at synonymous sites between species (*P. vivax* and *P. knowlesi*), d_S_ is the nucleotide diversity (polymorphism) within species (*P. vivax*), **μ**_s_ is synonymous mutation rate per generation and T*b* is time of the divergence between species (*P. vivax* and *P. knowlesi*). Effective population size of extant *P. vivax* was determined by the equation θ = 4*Ne* **μ** (for diploid organisms) and θ = 2*Ne* **μ** (for haploid and mitochondrial genomes), where θ (theta) is the mutation parameter, *Ne* the effective population size, and **μ** is the mutation rate per site per year.

### Bottleneck/stable population detection

Heterozygosity deficiency and mode shift analyses were used to detect evidence of a recent population bottleneck using BOTTLENECK software 1.2.02 [40]. In brief, a recently bottlenecked population would show higher observed gene diversity than the expected equilibrium gene diversity (Heq) which was computed from the observed number of alleles (k), under the assumption of a constant-size (equilibrium) population [41]. A two-phase mutation model (TPM) was used for heterozygosity deficiency analysis at mini- and microsatellite loci. TPM is an intermediate between the stepwise mutation model (SMM) and the infinite allele model (IAM), mostly consisting of one-step mutations having a small percentage (5–10%) of multi-step changes, as per BOTTLENECK software [41]. The allele frequency distribution analysis (mode shift analysis) showed whether it was approximately L-shaped (as expected under mutation-drift equilibrium) or not (recent bottlenecks provoke a mode shift). Bottleneck analysis can be performed using tandem repeat polymorphism data; the minisatellite polymorphism data being used in this study has been taken from earlier published work [28]. We also used network analysis of tandem repeat polymorphisms to detect possible signatures of bottlenecked or stable populations [42]. Two minisatellites located on a single chromosome (Chr 6), were used to construct reduced-median (RM) network of haplotypes using Network software [42]. In brief, a star-like network indicates a recent population expansion, whereas the absence of a star-like network indicates an ancient population expansion.

### Estimation of expected and observed pairwise differences

The estimation of expected and observed frequencies of pairwise differences at nucleotide sites provides a signature of whether a population is stable or has recently experienced a population bottleneck [36]. In summary, an L-shaped curve between observed and expected allele frequencies spectrum indicates a stable population whereas a non L-shaped curve suggests a population bottleneck. The pairwise nucleotide difference at individual housekeeping genes was estimated using DnaSP version 4.10 [32].

### Accession numbers

Accession numbers for alleles of all housekeeping genes are: HM047879–HM047972 (*ACPex*), HM047973–HM048020 (*ACPin*), HM048021–HM048117 (*DNA gyrase*), HM048118–HM048217 (*Exonuclease domain*), HM048118–HM048217 (*Enolase*), HM048318–HM048418 (*L34a*), HM048419–HM048518 (*L35e*), HM048619–HM048711 (*RNA polymerase-II*), HM048712–HM048811 (*Stpk*), and HM048812–HM048829 (*Ac*).

## Results

### Screening the genome for SNPs and tandem repeats

We screened the *P. vivax* Salvador 1 reference genome and identified ten putative housekeeping genes, and ten minisatellites and eight microsatellites, to infer the evolutionary history of this parasite in the Indian subcontinent. The selected housekeeping genes are *exonuclease domain (ed)*, *adenylate cyclase (ac)*, *serine/threonine protein kinase (stpk)*, *acyl carrier protein (acp)*, *ribosomal protein l35e (l35e)*, *60S ribosomal protein l34a (l34 a)*, *DNA gyrase (dg)*, *calcium-dependent protein kinase (cdpk)*, *enolase* and *RNA polymerase-II (Rpol)*. The structure of the housekeeping genes (arrangement of introns and exons), gene identifier and fragments used for SNP identification are given in **Figure 1** and **Table 1**. Approximately 400 to 600 bp of intron and exon sequence were used to identify SNPs. However, introns were not present in two housekeeping genes (*ac* and *ed*), therefore, partial exon sequence of these were used. The selected minisatellites and microsatellites are located at six different chromosomes and their features are listed in **Table S2**. Among the eight microsatellites, a single microsatellite (Gomez_1) was reported earlier by Gomez *et al* [43].

**Table 1:** Mutations in housekeeping genes from 100 Indian *Plasmodium vivax* isolates.

Housekeeping gene	Gene ID	Fragment size (bp)	Coding sites (bp)	Non-coding sites (bp)	Segregating sites	Synonymous substitution	Non-Synonymous substitution	Non-coding substitution	Total changes	Haplotype diversity (Hd)	Nucleotide diversity (π)
*Ribosomal protein l35e*	PVX_091865	518	170	348	12	2	5	5	13	0.665	0.00188
*Exonuclease Domain*	PVX_003660	300	300	0	1	1	0	0	1	0.366	0.00119
*Adenylate Cyclase*	PVX_092535	496	496	0	3	2	1	0	3	0.614	0.00146
*Acyl Carrier Protein*	PVX_114420	1336	706	630	10	3	3	4	10	0.494	0.00083
*Serine threonine protein kinase*	PVX_113435	1103	619	484	4	0	2	2	4	0.169	0.00016
*Ribosomal protein l34a*	PVX_088065	1134	586	548	7	0	0	7	7	0.864	0.00132
*RNA Polymerase-II*	PVX_122080	984	532	452	9	0	1	8	9	0.677	0.00106
*Enolase*	PVX_095015	1160	557	603	20	2	5	13	20	0.837	0.00147
*DNA Gyrase*	PVX_123795	1162	507	655	7	3	3	1	7	0.741	0.00092
*Calcium dependent protein kinase*	PVX_119610	1105	802	302	2	0	0	2	2	ND	ND
Total		9298	5275	4022	75	13	20	42	76		

ND: not determined.

### Putative housekeeping gene polymorphisms

Ten housekeeping genes were successfully amplified and sequenced to at least two-fold coverage. Analysis of the sequences revealed size polymorphisms in four of the housekeeping genes and two to six size variants were observed. These size variants were due to indels (*l35e*) or tandem repeat variation (*l34a, DNA gyrase*) in the gene's repeat region (**Figure S2**). Analyzing 9,298 nucleotide sites revealed a substantial number of SNPs (n = 75) in both coding and non-coding regions (Table 1). The number of synonymous, non-synonymous and non-coding SNPs observed, are listed in **Table 1**. On average, putative housekeeping genes had a low level of nucleotide (π = 0.002076±0.00059) and haplotype diversity (Hd = 0.5894±0.108) in *P. vivax* field isolates. Of the 75 SNPs identified, 55 were putatively selectively neutral (13 synonymous and 42 non-coding) and 20 were non-synonymous (**Table 1**). Sequencing of the *Pvcdpk* gene revealed presence of tandem repeats in the central region of gene, which caused difficulty in aligning the sequences correctly, and was therefore excluded from further analysis.

### Putative housekeeping genes are selectively neutral

The neutrality of housekeeping genes was inferred from the results of three tests. 1) The distribution of SNPs among exons (n = 33) and introns (n = 42) of putative housekeeping genes seems to be random (χ^2^ = 0.572, p = 0.449) suggesting a lack of functional constraint on exons. 2) By Tajima's D test, none of the putative housekeeping genes showed signs of significant deviation from neutrality (**Table 2**); however, Fu and Li's D test showed biased singleton mutation distribution in two of the nine putative housekeeping genes; these two genes were *enolase* (D = −2.920 p = <0.004) and *l35e* (D = −3.617, p = <0.004). 3) The d_N_/d_S_ ratio showed a signature of purifying selection for two putative housekeeping genes (*l35e* and *dg*), whereas mutations in the remaining genes were selectively neutral (**Table 2**). Further, none of the housekeeping genes showed significant departure from neutrality by the above three tests conducted together. Thus, these tests suggest that these housekeeping genes are primarily evolving in a neutral fashion and therefore can be employed as genetic markers for inferring population and evolutionary history of *P. vivax*.

**Table 2:** Neutrality test and mutation parameter of *Plasmodium vivax* housekeeping genes on the Indian subcontinent.

Gene	Tajima's D	Fu & Li's D	d_N_±SE (10^−3^)	d_S_±SE (10^−3^)	d_N_ = / = d_S_	Theta (θ) per site	Nucleotide substitutions rates in Pv		Divergence rates between Pv & Pk	
							dS	dN	Ks	Ka
*ed*	1.027	0.491	0.00	2.0±2.0	1.005	0.00064*	0.00	0.002±0.002	0.4334	0.1181
*ac*	−0.421	−0.804	7.0±5.0	0.00	−1.151	0.00173	0.00	0.001±0.001	0.3399	0.0410
*acp-in*	−0.434	1.006	**-**	-	-	0.00126	0.00	0.000	0.2907	0.0257
*acp-ex*	−1.36	−1.726	2.0±2.0	0.00	−0.955	0.00166	0.00	0.001±0.001	0.4202	0.1423
*l35e*	−1.543	**−2.92**	0.00	1.0±1.0	**2.074**	0.00448	0.004±0.003	0.000	0.3798	0.0134
*l34a*	0.231	−0.557	0.00	0.000	-	0.0012	0.00	0.0025±0.002	0.2332	0.0068
*stpk*	−1.487	−1.437	0.00	0.000	1.418	0.0007*	0.002±0.002	0.000	0.2575	0.0320
*enolase*	−1.624	**−3.617**	4.0±3.0	0.000	−1.035	0.00332	0.000	0.000	0.6643	0.5624
*RNA pol*	−1.031	−1.651	0.00	2.5±2.0	0.992	0.00179	0.007±0.005	0.000	0.5496	0.0867
*dg*	−0.501	−0.547	0.00	1.0±1.0	**2.061**	0.00117	-	-	0.6966	0.7130
Mean						0.002076±0.00059	0.004±0.001	0.0016±0.0003	0.3965	0.1142

**Boldface** values are statistically significant (p<0.05), *excluded in averaging **θ**, Pv: *Plasmodium vivax*, Pk: *P. knowlesi*, d_S_: synonymous substitution rate, d_N:_ non-synonymous substitution rate, Ks: synonymous divergence rate, Ka: non synonymous divergence rate.

### Lack of geographical structuring between populations

To understand whether *P. vivax* isolates are structured in populations, we undertook a genetic differentiation test using individual housekeeping genes. Our null hypothesis is that populations of *P. vivax* in the Indian subcontinent are genetically un-differentiated. Analysis of seven of the eight housekeeping genes accepted this hypothesis, while only *DNA gyrase* rejected the null hypothesis (**Table 3**). Since *DNA gyrase* does not appear to be neutral by neutrality tests (**Table 2**), it may not be ideal for measuring genetic differentiation between *P. vivax* populations. We confirmed the suitability of using chi-square statistics for genetic differentiation by determining the critical value of haplotype diversity (<0.95) [34]. The haplotype diversity measured at each housekeeping gene was lesser than the critical value (Hd = 0.169–0.864) (**Table 3**), confirming suitability of the use of chi-square statistics for genetic differentiation. Based on the seven selectively neutral housekeeping genes, it appears that different populations of *P. vivax* are not geographically structured in India. Thus, *P. vivax* isolates collected from different geographical regions of the Indian subcontinent will not have an adverse impact on the pooled analyses of TMRCA, effective population size, and population bottleneck.

**Table 3:** Genetic differentiation test between *Plasmodium vivax* populations in India.

Housekeeping genes	Sample size (N)	No of Haplotypes	Haplotype diversity (Hd = <0.95)	Chi square (χ^2^)	Degree of freedom	*P* value
*acpex*	95	7	0.425	25.241	24	0.392
*dg*	98	11	0.741	68.888	40	**0.003**
*ed*	100	2	0.366	4.291	4	0.368
*enolase*	100	22	0.837	104.361	84	0.065
*l34a*	100	10	0.864	51	36	0.053
*l35e*	100	14	0.665	68.006	52	0.067
*RNA pol-II*	94	12	0.677	48.65	44	0.291
*stpk*	100	5	0.169	12.709	12	0.39

Boldface values indicate significant genetic differentiation.

### Estimation of synonymous divergence rate and mutation rate

Since *P. vivax* is a close relative of *P. knowlesi* and the genome sequence of the latter has been determined [44], orthologous genes from *P. knowlesi* could be used to determine the genetic distance (divergence) between *P. knowlesi* and *P. vivax* and the pattern of evolutionary pressure (positive or negative selection) on these putative housekeeping genes. An approximately tenfold higher synonymous divergence rate over the non-synonymous divergence rate was observed between *P. vivax* and *P. knowlesi* (**Table 2**), suggesting that the putative housekeeping genes are evolving strictly under purifying selection. The average synonymous divergence and mutation rate for *P. vivax* was determined under various divergence time scenarios between the *P. knowlesi* and *P. vivax*. The estimated rates of synonymous divergence and mutation are given in **Table 4**. Based on this range, the synonymous divergence rate (per site per year) for the putative housekeeping genes was calculated as 8.61×10^−9^ (μ_sa_) and 6.60×10^−9^ (μ_sb_) under 23 mya and 30 mya divergence scenarios, respectively. Similarly, the mutation rates (per site per year) under the 23 mya and 30 mya divergence scenarios were determined to be 6.51×10^−9^ (μ_a_) and 4.99×10^−9^ (μ_b_), respectively. In contrast, few studies suggested relatively recent divergence time of *P. vivax* viz 2–3 mya [10] and 3.8–7 mya [13] and based on these divergence time mutation rates were also estimated. Under recent divergence time scenario, synonymous divergence rate (**μ**_s_) and mutation rate (**μ**) are calculated as above, and listed in **Table 4**.

**Table 4:** Estimation of mutation and synonymous divergence rates in *Plasmodium vivax*.

Divergence time scenario	Average mutation rate per site	Lower limits of mutation rate/site/year (μ_a_)	Upper limits of mutation rate/site/year (μ_b_)	Average synonymous divergence rate per site	Lower limits of synonymous divergence rate/site/year (μ_sa_)	Upper limits of synonymous divergence rate/site/year (μ_sb_)
[37] 23–30 mya	0.14985	6.51×10^−9^	4.99×10^−9^	0.3965	8.61×10^−9^	6.60×10^−9^
[39] 2–3 mya		7.49×10^−8^	4.99×10^−8^		9.90×10^−8^	6.60×10^−8^
[13] 3.8–6.3 mya		3.94×10^−8^	2.37×10^−8^		5.21×10^−8^	3.14×10^−8^

### TMRCA and effective population size (*Ne*) estimates

We determined a range of TMRCA for extant *P. vivax* in India that indicate for recent (20,202 to 30,303 years) or ancient (232,288 to 303,030 years) existence, based upon assumptions of recent or ancient divergence (**Table 5**). Similarly, on the basis of higher and lower mutation rate estimates, the effective population size estimate of extant *P. vivax* was obtained as recent (*Ne* = 6,929 to 10,400) and long-term (*Ne* = 79,235 to 104,008), respectively (**Table 4**).

**Table 5:** Time to Most Recent Common Ancestor (TMRCA) estimate of *Plasmodium vivax* on the Indian subcontinent.

Divergence time scenario	Synonymous substitutions rate (d_S_)	TMRCA lower limits (yrs)	TMRCA upper limits (yrs)	Theta (θ)	*Ne* lower limits	*Ne* upper limits	TMRCA estimates of *P. vivax* in India based on mt genome (yrs)		
							*Jongwutiwes et al. [14]	*Mu et al. [15]	#Cornejo et al. [50]
23–30 mya	0.004±0.001	232288±58072	303030±75757	0.002076±0.00059	79235±22657	104008±29559	13,000–17,000	122,700–164,900	143 400–192 600
3.8–6.3 mya		38387±9596	63897±15923		13172±3747	21898±6223			
2–3 mya		20202±5050	30303±7575		6929±1969	10400±2933			

* : The TMRCA of Indian *P. vivax* is estimated by Cornejo et al, #: TMRCA estimates analyzed by joining data of Jongwutiwe et al 2005, and Mu et al 2005.

### *Plasmodium vivax* populations are stable

**Figure 2:**
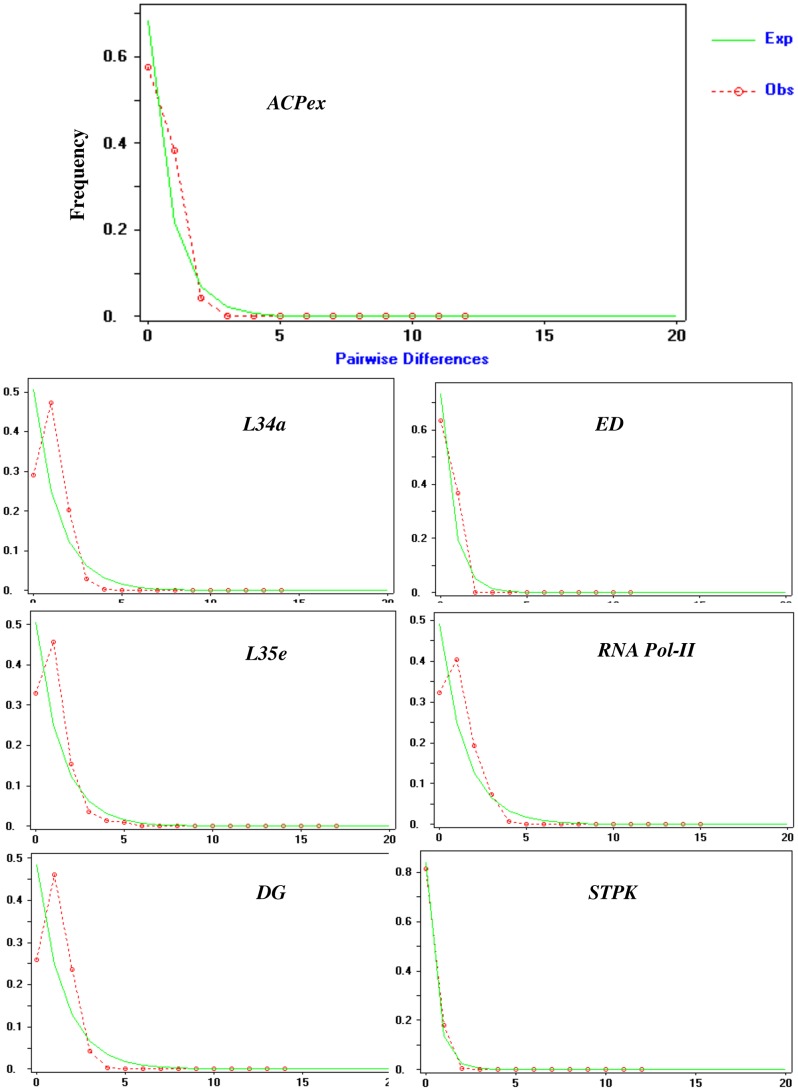
Expected and observed pairwise differences at seven *Plasmodium vivax* housekeeping genes.

We tested the housekeeping gene polymorphism data to see if there were detectable differences between the observed and expected allele frequency, which might indicate an unstable population structure of *P. vivax* in India. Assuming constant population equilibrium, we determined that the observed allele frequency spectrum was similar to the expected one (**Figure 2**). In addition, we calculated the pairwise difference analysis for all samples and individual populations (**Figure S3**), and also found the observed allele frequency spectrum followed the expected frequency. Thus this analysis indicates the stable nature of *P. vivax* populations in the Indian subcontinent.

### Tandem repeat variability and population bottleneck

**Figure 3:**
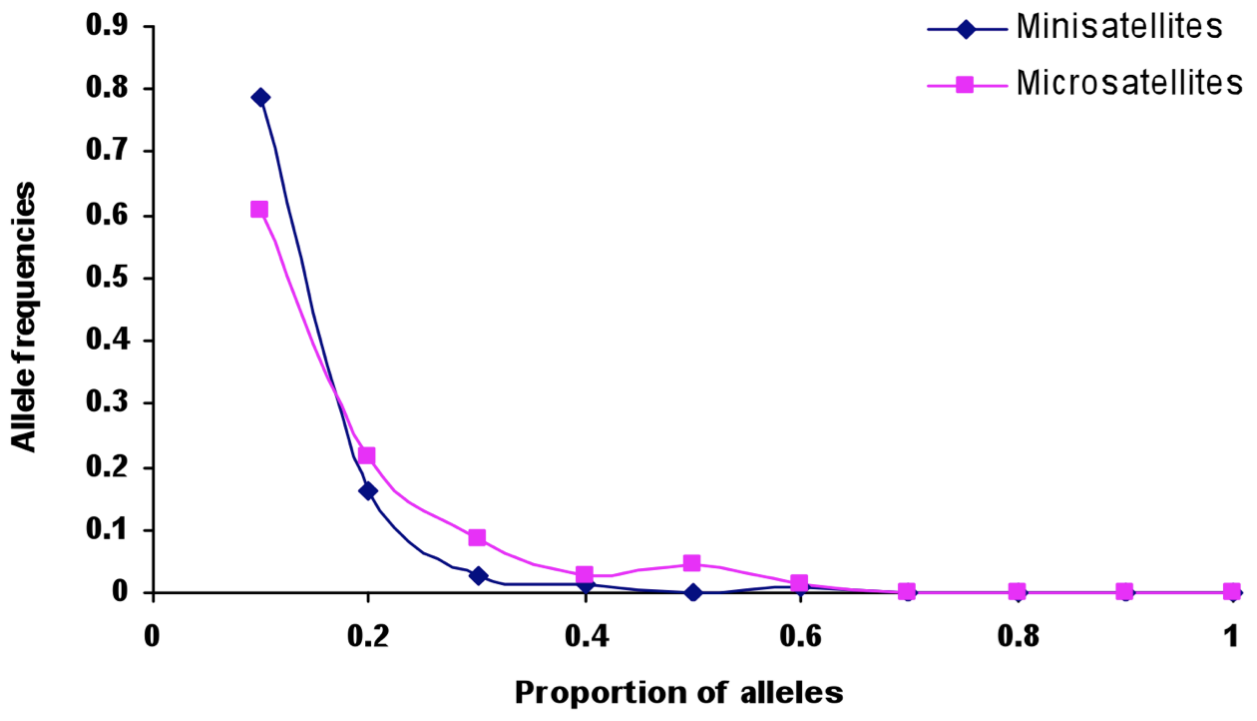
Allele frequency distribution curve based on *Plasmodium vivax* minisatellite and microsatellite polymorphism in the Indian subcontinent.

Seven microsatellites, three from one chromosome (Chr 6), one each from four chromosomes (Chr 2, Chr 5, Chr 7 and Chr10), were identified from the *P. vivax* genome sequence, and a subset of isolates (n = 40) was tested by microsatellite analysis. A substantial number of alleles (range 6–13, *AE* = 8.62±1.16) and high expected heterozygosity (*He* = 0.65 to 0.90, *AE* = 0.789±0.039) were observed for each locus. Using data that we had previously generated for ten minisatellites on the same sample set used here [28], bottleneck analysis (heterozygote deficiency and allele frequency mode shift tests) was undertaken to detect whether the extant *P. vivax* population in the Indian subcontinent reflects a signature of a recent population bottleneck or of a more ancient population. A majority of loci (80%) showed heterozygosity deficiency (**Table 6**), suggesting that they are in expected genetic diversity equilibrium. Similarly, mode shift analysis revealed a strictly L-shaped allele frequency distribution (**Figure 3**). The high heterozygosity deficiency and L-shaped allele frequency distribution at minisatellites suggests that the Indian *P. vivax* population has not undergone a recent population bottleneck. This supports our ancient age estimates of *P. vivax* inferred from putative housekeeping genes.

**Table 6:** Observed and expected heterozygosity at mini and microsatellite loci of *Plasmodium vivax* on the Indian subcontinent.

Locus	Observed (*He*)			Calculated by TPM (*Heq*)		
	N	K	*He*	*Heq*	SD	DH/sd
PvCDPK	95	22	0.916	**0.932**	0.013	−1.254
MiniSat 1	95	18	0.826	**0.911**	0.017	−4.994
MiniSat 2	92	11	0.842	**0.84**	0.038	0.074
MiniSat 5	93	16	0.912	0.898	0.023	0.616
MiniSat 6	95	16	0.888	**0.897**	0.021	−0.426
MiniSat 8	55	7	0.698	**0.757**	0.067	−0.876
MiniSat 11	94	18	0.924	0.91	0.02	0.7
MiniSat 13	91	14	0.887	**0.88**	0.025	0.279
MiniSat 14	96	14	0.873	**0.878**	0.027	−0.161
MiniSat 16	95	11	0.815	**0.838**	0.038	−0.582
MS_38	36	6	0.656	**0.735**	0.073	−1.088
MS_40	40	7	0.786	0.772	0.062	0.223
MS_50	39	9	0.779	**0.833**	0.041	−1.327
MS_21	39	8	0.734	**0.808**	0.050	−1.497
MS_73	40	7	0.836	0.772	0.060	1.061
MS_92	37	11	0.862	**0.876**	0.031	−0.472
MS_128	38	13	0.905	**0.900**	0.035	0.150
Gomez_1	40	8	0.760	**0.808**	0.052	−0.922

N: number of samples, K: number of alleles observed, *He*: observed gene diversity and *Heq*: expected gene diversity equilibrium. **Boldface** values indicate heterozygosity deficiency.

### Network analysis of tandem repeat loci

**Figure 4:**
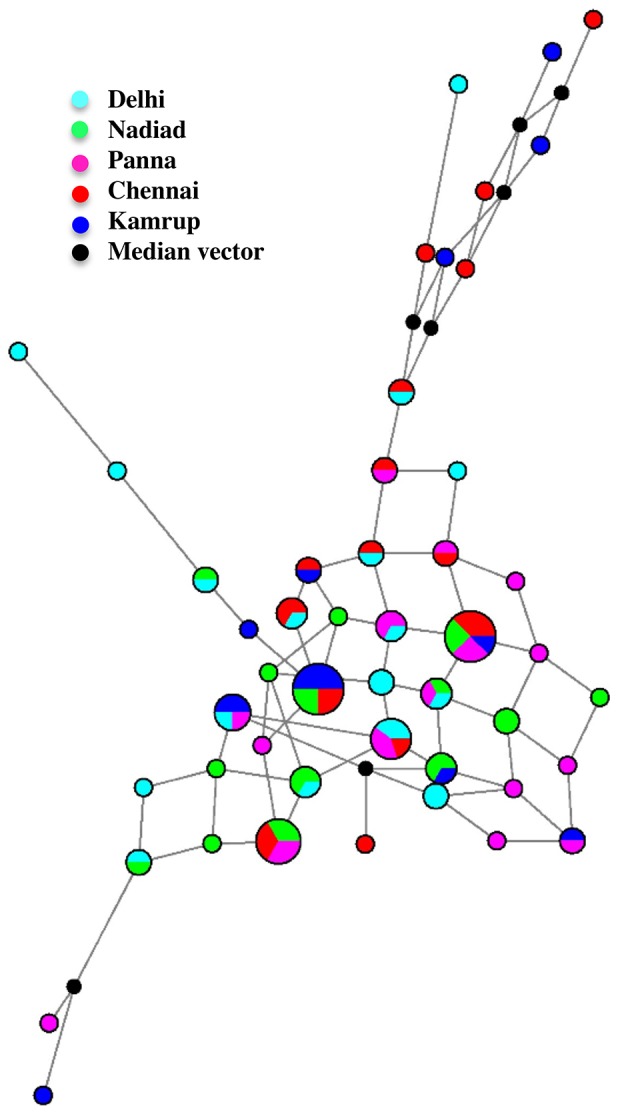
Reduced-median haplotype network derived from *Plasmodium vivax* tandem repeat loci.

We constructed a reduced-median (RM) network of tandem repeat-based haplotypes to understand whether *P. vivax* populations in India expanded recently (which would produce a star-like network). A RM haplotype network derived from more than two tandem repeat loci produces a highly complex network, therefore, we limited our analysis to two tandem repeat loci on *P. vivax* Chr 6. No star- like network of haplotypes was produced (**Figure 4**), suggesting an ancient population expansion of *P. vivax* has occurred in the Indian subcontinent.

## Discussion

This is the first study concerning *P. vivax* evolutionary history on the Indian subcontinent using SNPs in putative housekeeping genes and minisatellite and microsatellite markers. In this study we identified 1) polymorphisms in Indian *P. vivax* populations at neutral genetic loci; 2) neutral housekeeping genes suitable for inferring the evolutionary history of *P. vivax* in India; 3) no geographical structuring of *P. vivax* populations; and 4) an ancient and stable population of *P. vivax* on the Indian subcontinent.

The high degree of genetic polymorphism observed in tandem repeat loci agrees with earlier studies which revealed tremendous genetic polymorphism among global *P. vivax* isolates [22], [43], [45], [46], [47], [48]. This study successfully identified neutral genetic variation in several genetic loci, and this renders them highly useful as molecular markers for population structure studies. The high density of putatively neutral SNPs observed in housekeeping genes among Indian *P. vivax* isolates is in accordance with observations of Feng *et al.* [22], suggesting SNPs in *P. vivax* isolates are comparatively more common than in *P. falciparum* [17], [22]. Recently this was confirmed by deep sequencing that showed a higher genetic variability in *P. vivax* than *P. falciparum* [49]. Our neutrality test results imply that most of the housekeeping genes are free from directed selection pressure. Neutral evolution of the housekeeping genes described here argues for their utility as molecular markers for understanding demographic events and population history of *P. vivax*. We also tested whether pooling the *P. vivax* isolates collected from five geographically disparate populations might have adversely impacted the analyses of TMRCA and effective population size estimations, due to geographic structuring of subpopulations. However, we found no evidence for significant divergence between the populations, suggest that pooling of samples will not have an adverse impact on the analyses of TMRCA and effective population size. The absence of population structure between these five populations was also confirmed in an earlier study on the basis of genetic distance data derived from ten minisatellite loci [28].

Using the neutral polymorphisms of several housekeeping genes, we deduced that the MRCA of extant *P. vivax* was present on the Indian subcontinent about 232,228 to 303,030 years ago. This ancient TMRCA is strongly supported by long-term effective *P. vivax* population size (*Ne*) estimates and stable population indexes. The stable population index of *P. vivax* in Indian populations was established earlier using other neutral genetic loci [26] that also supports our older age estimate of this species. This ancient evolutionary history of extant *P. vivax* in India is similar to the molecular age inferred from mitochondrial genome diversity, *i.e.*, 162,400–464,600 years [14], [15], [50]. Our TMRCA estimate strongly supports the hypothesis that *P. vivax* was a hominoid primate parasite before it became a human parasite by means of a host switch [12], [15], [50] and that it has evolved in Southeast Asia. The alternative hypothesis claiming an African origin for *P. vivax* is based on the fixation of the Duffy negative trait in the black African population. However, this alternate hypothesis is fraught with inconsistencies. For example, the Duffy negative trait arose around 100,000 years ago [51]; this time scale is shorter than the *P. vivax* TMRCA estimated in the present study and others [14], [15], [50]. The Duffy receptor is used for invasion by many pathogens including bacteria [52] and viruses [53], [54], [55]; thus, *P. vivax* is not necessarily the only evolutionary force for the fixation of this trait in the West African native population. Finally, *P. vivax* infection of Duffy negative people [56], [57], [58] suggests the existence of alternate invasion mechanisms, such as occur in *P. falciparum* [59], [60], [61].

In contrast, a much younger age estimate of *P. vivax* was determined under a recent divergence model of *P. vivax* and *P. cynomolgi* [10] coincident with the radiation of Asian macaques 2–3 mya [39]. This assumption implies that the MRCA of extant *P. vivax* dates to just 20,202–30,303 years ago. It is of interest to mention that under this recent divergence model [10], *P. vivax* shows a ten-fold higher mutation rate than *P. falciparum* [17], and it is unlikely that such a high mutation rate difference exists between related species. The radiation of Asian macaques was inferred from analysis of a mitochondrial gene, which may be evolving at a different rate than nuclear genes. Combined, our population genetic analyses such as pairwise difference, allele frequency, reduced-median network of haplotypes, and heterozygote deficiency conducted in the present study do not reflect the signature of a recent population expansion or bottlenecked population of *P. vivax* in the Indian subcontinent. Therefore, an ancient divergence time (23–30 mya) of *P. vivax* and *P. knowlesi*, seems more plausible.

In conclusion, our findings reveal that *P. vivax* isolates have an ancient evolutionary history in the Indian subcontinent. For the first time we report that neutral nuclear genome markers (housekeeping genes) displayed an evolutionary history of *P. vivax* similar to that inferred from mitochondrial genome diversity.

## Supporting Information

Figure S1 Map of India showing location of *Plasmodium vivax* sampling sites. 1: Delhi; 2: Panna, Madhya Pradesh; 3: Nadiad, Gujarat; 4: Chennai, Tamil Nadu; and 5: Kamrup, Assam.
(PPT)

Figure S2 Tandem repeat variation in housekeeping genes *from Plasmodium vivax* field isolates. A) *DNA gyrase* and B) *Ribosomal protein l34a*. Tandem repeat unit is underlined. Dash (–) represents deleted nucleotides.
(PPT)

Figure S3 Expected and observed pairwise differences at seven *Plasmodium vivax* housekeeping genes.
(PPT)

Table S1 Primers and amplification conditions.
(DOC)

Table S2 Characteristic features of *Plasmodium vivax* mini and microsatellite markers.
(DOC)

Text S1 Details of study sites.
(DOC)

## References

[1] Andrade BB, Reis-Filho A, Souza-Neto SM, Clarencio J, Camargo LM, et al (2010) Severe Plasmodium vivax malaria exhibits marked inflammatory imbalance. Malar J 9: 13. 20070895 10.1186/1475-2875-9-13 PMC2837053

[2] Kochar DK, Das A, Kochar SK, Saxena V, Sirohi P, et al (2009) Severe Plasmodium vivax malaria: a report on serial cases from Bikaner in northwestern India. Am J Trop Med Hyg 80: 194–198. 19190212

[3] Kochar DK, Saxena V, Singh N, Kochar SK, Kumar SV, et al (2005) Plasmodium vivax malaria. Emerg Infect Dis 11: 132–134. 15705338 10.3201/eid1101.040519 PMC3294370

[4] Genton B, D'Acremont V, Rare L, Baea K, Reeder JC, et al (2008) Plasmodium vivax and mixed infections are associated with severe malaria in children: a prospective cohort study from Papua New Guinea. PLoS Med 5: e127. 18563961 10.1371/journal.pmed.0050127 PMC2429951

[5] Rogerson SJ, Carter R (2008) Severe vivax malaria: newly recognised or rediscovered. PLoS Med 5: e136. 18563965 10.1371/journal.pmed.0050136 PMC2429947

[6] Tjitra E, Anstey NM, Sugiarto P, Warikar N, Kenangalem E, et al (2008) Multidrug-resistant Plasmodium vivax associated with severe and fatal malaria: a prospective study in Papua, Indonesia. PLoS Med 5: e128. 18563962 10.1371/journal.pmed.0050128 PMC2429950

[7] Miller LH, Mason SJ, Clyde DF, McGinniss MH (1976) The resistance factor to Plasmodium vivax in blacks. The Duffy-blood-group genotype, FyFy. N Engl J Med 295: 302–304. 778616 10.1056/NEJM197608052950602

[8] Escalante AA, Ayala FJ (1994) Phylogeny of the malarial genus Plasmodium, derived from rRNA gene sequences. Proc Natl Acad Sci U S A 91: 11373–11377. 7972067 10.1073/pnas.91.24.11373 PMC45233

[9] Escalante AA, Ayala FJ (1995) Evolutionary origin of Plasmodium and other Apicomplexa based on rRNA genes. Proc Natl Acad Sci U S A 92: 5793–5797. 7597031 10.1073/pnas.92.13.5793 PMC41587

[10] Escalante AA, Freeland DE, Collins WE, Lal AA (1998) The evolution of primate malaria parasites based on the gene encoding cytochrome b from the linear mitochondrial genome. Proc Natl Acad Sci U S A 95: 8124–8129. 9653151 10.1073/pnas.95.14.8124 PMC20940

[11] Waters AP, Higgins DG, McCutchan TF (1993) Evolutionary relatedness of some primate models of Plasmodium. Mol Biol Evol 10: 914–923. 7689135 10.1093/oxfordjournals.molbev.a040038

[12] Escalante AA, Cornejo OE, Freeland DE, Poe AC, Durrego E, et al (2005) A monkey's tale: the origin of Plasmodium vivax as a human malaria parasite. Proc Natl Acad Sci U S A 102: 1980–1985. 15684081 10.1073/pnas.0409652102 PMC548581

[13] Hayakawa T, Culleton R, Otani H, Horii T, Tanabe K (2008) Big bang in the evolution of extant malaria parasites. Mol Biol Evol 25: 2233–2239. 18687771 10.1093/molbev/msn171

[14] Jongwutiwes S, Putaporntip C, Iwasaki T, Ferreira MU, Kanbara H, et al (2005) Mitochondrial genome sequences support ancient population expansion in Plasmodium vivax. Mol Biol Evol 22: 1733–1739. 15901839 10.1093/molbev/msi168 PMC1224720

[15] Mu J, Joy DA, Duan J, Huang Y, Carlton J, et al (2005) Host switch leads to emergence of Plasmodium vivax malaria in humans. Mol Biol Evol 22: 1686–1693. 15858201 10.1093/molbev/msi160

[16] Rich SM, Licht MC, Hudson RR, Ayala FJ (1998) Malaria's Eve: evidence of a recent population bottleneck throughout the world populations of Plasmodium falciparum. Proc Natl Acad Sci U S A 95: 4425–4430. 9539753 10.1073/pnas.95.8.4425 PMC22505

[17] Volkman SK, Barry AE, Lyons EJ, Nielsen KM, Thomas SM, et al (2001) Recent origin of Plasmodium falciparum from a single progenitor. Science 293: 482–484. 11463913 10.1126/science.1059878

[18] Tanabe K, Sakihama N, Hattori T, Ranford-Cartwright L, Goldman I, et al (2004) Genetic distance in housekeeping genes between Plasmodium falciparum and Plasmodium reichenowi and within P. falciparum. J Mol Evol 59: 687–694. 15693624 10.1007/s00239-004-2662-3

[19] Anderson TJ, Nair S, Sudimack D, Williams JT, Mayxay M, et al (2005) Geographical distribution of selected and putatively neutral SNPs in Southeast Asian malaria parasites. Mol Biol Evol 22: 2362–2374. 16093566 10.1093/molbev/msi235

[20] Anderson TJ, Su XZ, Bockarie M, Lagog M, Day KP (1999) Twelve microsatellite markers for characterization of Plasmodium falciparum from finger-prick blood samples. Parasitology 119 (Pt 2) 113–125. 10466118 10.1017/s0031182099004552

[21] Mu J, Duan J, Makova KD, Joy DA, Huynh CQ, et al (2002) Chromosome-wide SNPs reveal an ancient origin for Plasmodium falciparum. Nature 418: 323–326. 12124624 10.1038/nature00836

[22] Feng X, Carlton JM, Joy DA, Mu J, Furuya T, et al (2003) Single-nucleotide polymorphisms and genome diversity in Plasmodium vivax. Proc Natl Acad Sci U S A 100: 8502–8507. 12799466 10.1073/pnas.1232502100 PMC166258

[23] Carlton JM, Adams JH, Silva JC, Bidwell SL, Lorenzi H, et al (2008) Comparative genomics of the neglected human malaria parasite Plasmodium vivax. Nature 455: 757–763. 18843361 10.1038/nature07327 PMC2651158

[24] Carlton JM, Escalante AA, Neafsey D, Volkman SK (2008) Comparative evolutionary genomics of human malaria parasites. Trends Parasitol 24: 545–550. 18938107 10.1016/j.pt.2008.09.003

[25] Kumar A, Valecha N, Jain T, Dash AP (2007) Burden of malaria in India: retrospective and prospective view. Am J Trop Med Hyg 77: 69–78. 18165477

[26] Gupta B, Srivastava N, Das A (2012) Inferring the evolutionary history of Indian Plasmodium vivax from population genetic analyses of multilocus nuclear DNA fragments. Mol Ecol 21: 1597–1616. 22353169 10.1111/j.1365-294X.2012.05480.x

[27] Chittoria A, Mohanty S, Jaiswal YK, Das A (2012) Natural selection mediated association of the Duffy (FY) gene polymorphisms with Plasmodium vivax malaria in India. PLoS One 7: e45219. 23028857 10.1371/journal.pone.0045219 PMC3448599

[28] Prajapati SK, Joshi H, Shalini S, Patarroyo MA, Suwanarusk R, et al (2011) Plasmodium vivax lineages: geographical distribution, tandem repeat polymorphism, and phylogenetic relationship. Malar J 10: 374. 22182774 10.1186/1475-2875-10-374 PMC3258263

[29] Prajapati SK, Kumari P, Singh OP (2012) Molecular analysis of reticulocyte binding protein-2 gene in Plasmodium vivax isolates from India. BMC Microbiol 12: 243. 23096021 10.1186/1471-2180-12-243 PMC3539960

[30] Bruce MC, Galinski MR, Barnwell JW, Snounou G, Day KP (1999) Polymorphism at the merozoite surface protein-3alpha locus of Plasmodium vivax: global and local diversity. Am J Trop Med Hyg 61: 518–525. 10548283 10.4269/ajtmh.1999.61.518

[31] Benson G (1999) Tandem repeats finder: a program to analyze DNA sequences. Nucleic Acids Res 27: 573–580. 9862982 10.1093/nar/27.2.573 PMC148217

[32] Rozas J, Sanchez-DelBarrio JC, Messeguer X, Rozas R (2003) DnaSP, DNA polymorphism analyses by the coalescent and other methods. Bioinformatics 19: 2496–2497. 14668244 10.1093/bioinformatics/btg359

[33] Dieringer D, Schlötterer C (2003) Microsatellite analyser (MSA): a platform independent analysis tool for large microsatellite data sets. Molecular Ecology Notes 3: 168–169.

[34] Nei M (1987) Molecular evolutionary genetics. New York: Columbia University Press.

[35] Fu XY, Li WH (1993) Statistical tests of neutrality of mutations. Genetics 133. 10.1093/genetics/133.3.693 PMC1205353 8454210

[36] Tajima F (1989) Statistical method for testing the neutral mutation hypothesis by DNA polymorphism. Genetics 123: 585–595. 2513255 10.1093/genetics/123.3.585 PMC1203831

[37] Kumar S, Hedges SB (1998) A molecular timescale for vertebrate evolution. Nature 392: 917–920. 9582070 10.1038/31927

[38] Sibley CG, Ahlquist JE (1987) DNA hybridization evidence of hominoid phylogeny: results from an expanded data set. J Mol Evol 26: 99–121. 3125341 10.1007/BF02111285

[39] Hayasaka K, Fujii K, Horai S (1996) Molecular phylogeny of macaques: implications of nucleotide sequences from an 896-base pair region of mitochondrial DNA. Mol Biol Evol 13: 1044–1053. 8752012 10.1093/oxfordjournals.molbev.a025655

[40] Cornuet JM, Luikart G (1996) Description and power analysis of two tests for detecting recent population bottlenecks from allele frequency data. Genetics 144: 2001–2014. 8978083 10.1093/genetics/144.4.2001 PMC1207747

[41] Luikart G, Allendorf FW, Cornuet JM, Sherwin WB (1998) Distortion of allele frequency distributions provides a test for recent population bottlenecks. J Hered 89: 238–247. 9656466 10.1093/jhered/89.3.238

[42] Bandelt HJ, Forster P, Sykes BC, Richards MB (1995) Mitochondrial portraits of human populations using median networks. Genetics 141: 743–753. 8647407 10.1093/genetics/141.2.743 PMC1206770

[43] Gomez JC, McNamara DT, Bockarie MJ, Baird JK, Carlton JM, et al (2003) Identification of a polymorphic Plasmodium vivax microsatellite marker. Am J Trop Med Hyg 69: 377–379. 14640496 PMC3728893

[44] Pain A, Bohme U, Berry AE, Mungall K, Finn RD, et al (2008) The genome of the simian and human malaria parasite Plasmodium knowlesi. Nature 455: 799–803. 18843368 10.1038/nature07306 PMC2656934

[45] Imwong M, Nair S, Pukrittayakamee S, Sudimack D, Williams JT, et al (2007) Contrasting genetic structure in Plasmodium vivax populations from Asia and South America. Int J Parasitol 37: 1013–1022. 17442318 10.1016/j.ijpara.2007.02.010

[46] Imwong M, Snounou G, Pukrittayakamee S, Tanomsing N, Kim JR, et al (2007) Relapses of Plasmodium vivax infection usually result from activation of heterologous hypnozoites. J Infect Dis 195: 927–933. 17330781 10.1086/512241

[47] Imwong M, Sudimack D, Pukrittayakamee S, Osorio L, Carlton JM, et al (2006) Microsatellite variation, repeat array length, and population history of Plasmodium vivax. Mol Biol Evol 23: 1016–1018. 16507919 10.1093/molbev/msj116

[48] Karunaweera ND, Ferreira MU, Munasinghe A, Barnwell JW, Collins WE, et al (2008) Extensive microsatellite diversity in the human malaria parasite Plasmodium vivax. Gene 410: 105–112. 18226474 10.1016/j.gene.2007.11.022

[49] Neafsey DE, Galinsky K, Jiang RH, Young L, Sykes SM, et al (2012) The malaria parasite Plasmodium vivax exhibits greater genetic diversity than Plasmodium falciparum. Nat Genet 44: 1046–1050. 22863733 10.1038/ng.2373 PMC3432710

[50] Cornejo OE, Escalante AA (2006) The origin and age of Plasmodium vivax. Trends Parasitol 22: 558–563. 17035086 10.1016/j.pt.2006.09.007 PMC1855252

[51] Hamblin MT, Di Rienzo A (2000) Detection of the signature of natural selection in humans: evidence from the Duffy blood group locus. Am J Hum Genet 66: 1669–1679. 10762551 10.1086/302879 PMC1378024

[52] Cundell DR, Gerard NP, Gerard C, Idanpaan-Heikkila I, Tuomanen EI (1995) Streptococcus pneumoniae anchor to activated human cells by the receptor for platelet-activating factor. Nature 377: 435–438. 7566121 10.1038/377435a0

[53] He W, Neil S, Kulkarni H, Wright E, Agan BK, et al (2008) Duffy antigen receptor for chemokines mediates trans-infection of HIV-1 from red blood cells to target cells and affects HIV-AIDS susceptibility. Cell Host Microbe 4: 52–62. 18621010 10.1016/j.chom.2008.06.002 PMC2562426

[54] Doranz BJ, Grovit-Ferbas K, Sharron MP, Mao SH, Goetz MB, et al (1997) A small-molecule inhibitor directed against the chemokine receptor CXCR4 prevents its use as an HIV-1 coreceptor. J Exp Med 186: 1395–1400. 9334380 10.1084/jem.186.8.1395 PMC2199097

[55] Fauci AS (1996) Host factors and the pathogenesis of HIV-induced disease. Nature 384: 529–534. 8955267 10.1038/384529a0

[56] Menard D, Barnadas C, Bouchier C, Henry-Halldin C, Gray LR, et al (2010) Plasmodium vivax clinical malaria is commonly observed in Duffy-negative Malagasy people. Proc Natl Acad Sci U S A 107: 5967–5971. 20231434 10.1073/pnas.0912496107 PMC2851935

[57] Ryan JR, Stoute JA, Amon J, Dunton RF, Mtalib R, et al (2006) Evidence for transmission of Plasmodium vivax among a duffy antigen negative population in Western Kenya. Am J Trop Med Hyg 75: 575–581. 17038676

[58] Cavasini CE, Mattos LC, Couto AA, Bonini-Domingos CR, Valencia SH, et al (2007) Plasmodium vivax infection among Duffy antigen-negative individuals from the Brazilian Amazon region: an exception? Trans R Soc Trop Med Hyg 101: 1042–1044. 17604067 10.1016/j.trstmh.2007.04.011

[59] Baum J, Maier AG, Good RT, Simpson KM, Cowman AF (2005) Invasion by P. falciparum merozoites suggests a hierarchy of molecular interactions. PLoS Pathog 1: e37. 16362075 10.1371/journal.ppat.0010037 PMC1315277

[60] Dolan SA, Miller LH, Wellems TE (1990) Evidence for a switching mechanism in the invasion of erythrocytes by Plasmodium falciparum. J Clin Invest 86: 618–624. 2200806 10.1172/JCI114753 PMC296769

[61] Prajapati SK, Singh OP (2013) Insights into the invasion biology of Plasmodium vivax. Front Cell Infect Microbiol 3: 8. 23469364 10.3389/fcimb.2013.00008 PMC3587795

