# Supplementary material for: Neutral Polymorphisms in Putative Housekeeping Genes and Tandem Repeats Unravels the Population Genetics and Evolutionary History of Plasmodium vivax in India
Source: PLoS Negl Trop Dis. 2013 Sep 19;7(9):e2425. doi: 10.1371/journal.pntd.0002425 (PMC3777877; doi:10.1371/journal.pntd.0002425)
Supplement: Figure S1 — Map of India showing location of Plasmodium vivax sampling sites. 1: Delhi; 2: Panna, Madhya Pradesh; 3: Nadiad, Gujarat; 4: Chennai, Tamil Nadu; and 5: Kamrup, Assam. (PPT) [file pntd.0002425.s001.ppt]

## Slide 1
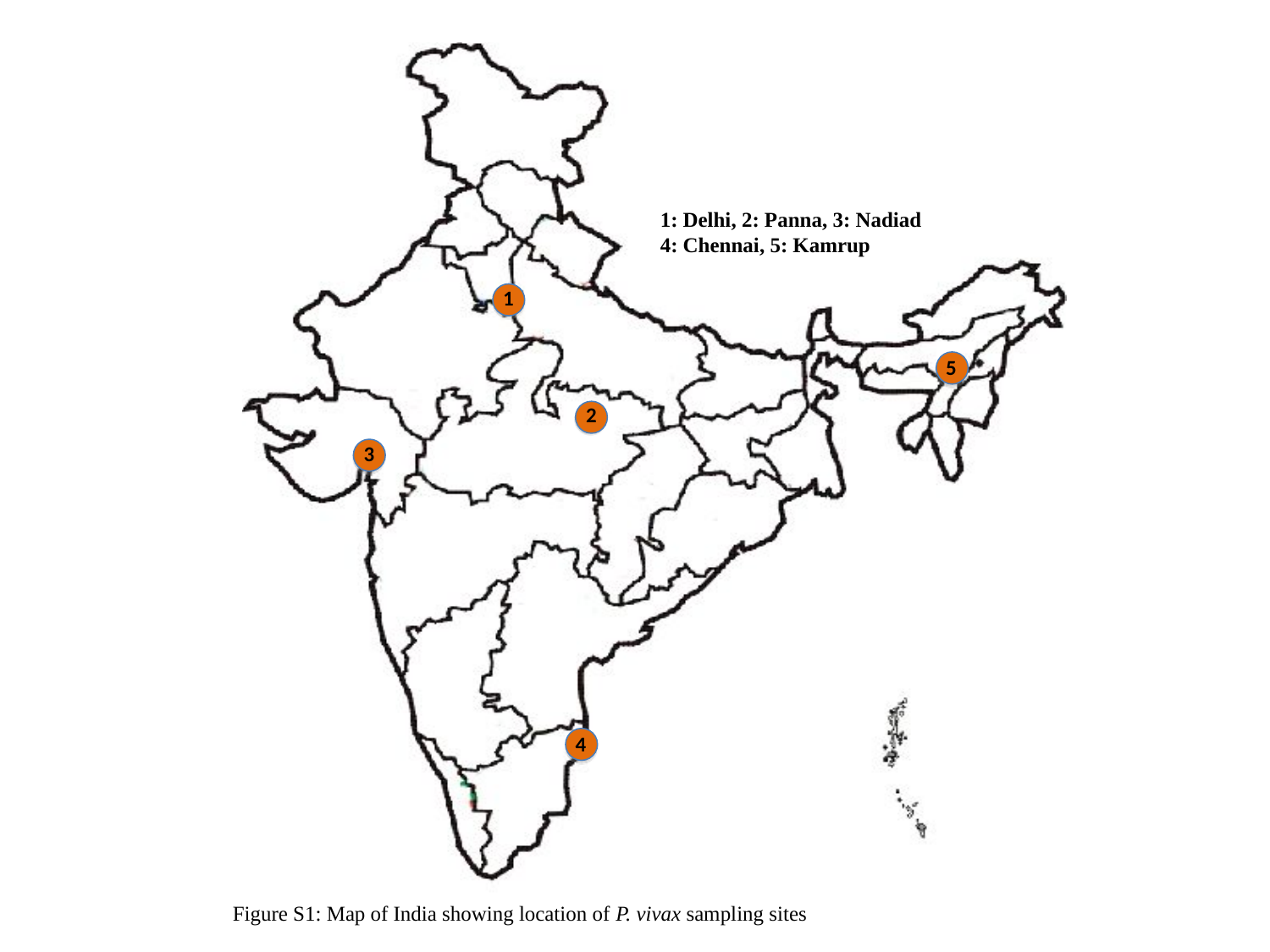

1: Delhi, 2: Panna, 3: Nadiad
4: Chennai, 5: Kamrup
1
5
2
3
4
Figure S1: Map of India showing location of P. vivax sampling sites
