# Supplementary material for: Neutral Polymorphisms in Putative Housekeeping Genes and Tandem Repeats Unravels the Population Genetics and Evolutionary History of Plasmodium vivax in India
Source: PLoS Negl Trop Dis. 2013 Sep 19;7(9):e2425. doi: 10.1371/journal.pntd.0002425 (PMC3777877; doi:10.1371/journal.pntd.0002425)
Supplement: Figure S2 — Tandem repeat variation in housekeeping genes from Plasmodium vivax field isolates. A) DNA gyrase and B) Ribosomal protein l34a. Tandem repeat unit is underlined. Dash (–) represents deleted nucleotides. (PPT) [file pntd.0002425.s002.ppt]

## Slide 1
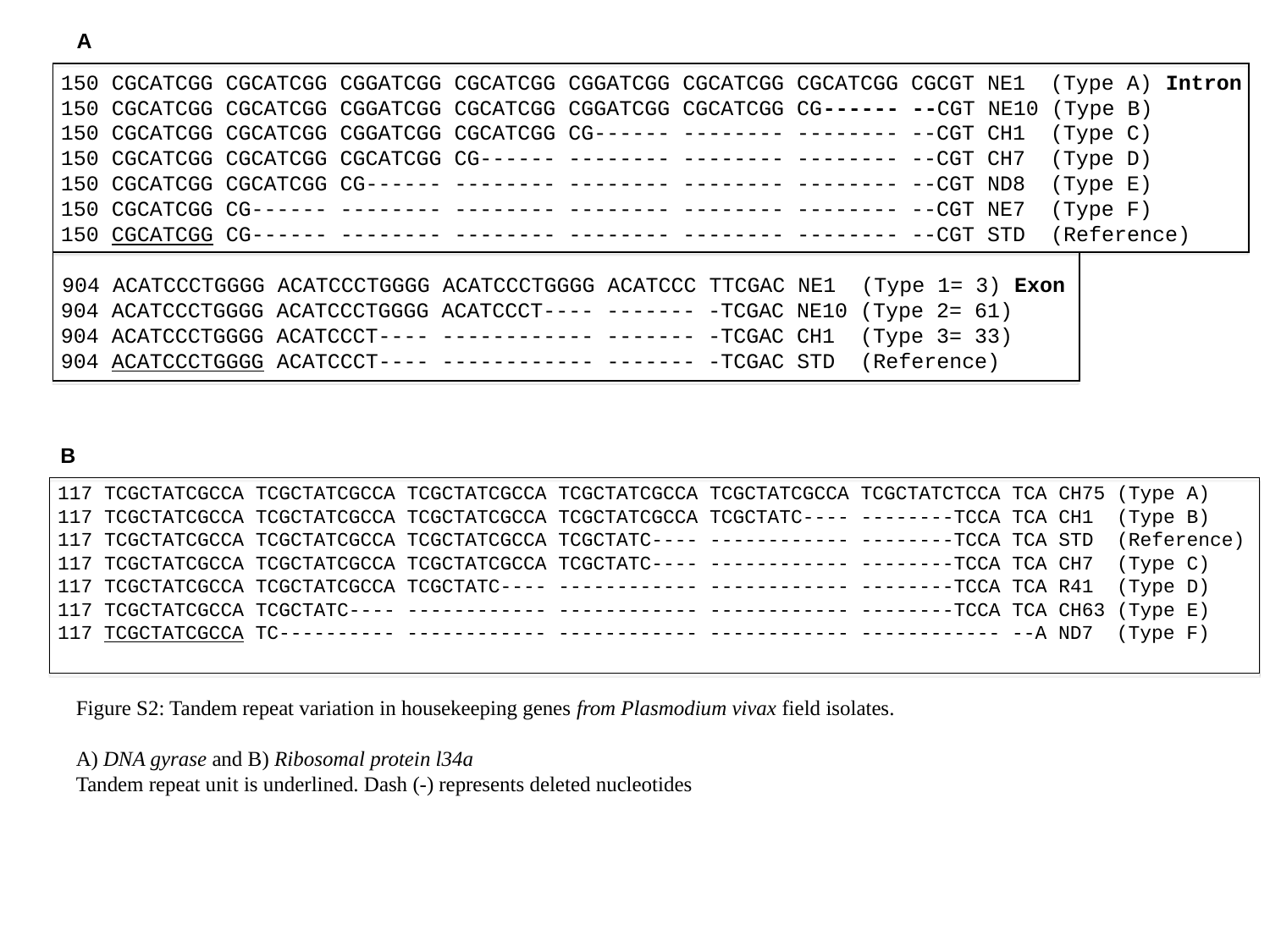

A
 150 CGCATCGG CGCATCGG CGGATCGG CGCATCGG CGGATCGG CGCATCGG CGCATCGG CGCGT NE1 (Type A) Intron
 150 CGCATCGG CGCATCGG CGGATCGG CGCATCGG CGGATCGG CGCATCGG CG------ --CGT NE10 (Type B)
 150 CGCATCGG CGCATCGG CGGATCGG CGCATCGG CG------ -------- -------- --CGT CH1 (Type C)
 150 CGCATCGG CGCATCGG CGCATCGG CG------ -------- -------- -------- --CGT CH7 (Type D)
 150 CGCATCGG CGCATCGG CG------ -------- -------- -------- -------- --CGT ND8 (Type E)
 150 CGCATCGG CG------ -------- -------- -------- -------- -------- --CGT NE7 (Type F)
 150 CGCATCGG CG------ -------- -------- -------- -------- -------- --CGT STD (Reference)
 904 ACATCCCTGGGG ACATCCCTGGGG ACATCCCTGGGG ACATCCC TTCGAC NE1 (Type 1= 3) Exon
 904 ACATCCCTGGGG ACATCCCTGGGG ACATCCCT---- ------- -TCGAC NE10 (Type 2= 61)
 904 ACATCCCTGGGG ACATCCCT---- ------------ ------- -TCGAC CH1 (Type 3= 33)
 904 ACATCCCTGGGG ACATCCCT---- ------------ ------- -TCGAC STD (Reference)
B
 117 TCGCTATCGCCA TCGCTATCGCCA TCGCTATCGCCA TCGCTATCGCCA TCGCTATCGCCA TCGCTATCTCCA TCA CH75 (Type A)
 117 TCGCTATCGCCA TCGCTATCGCCA TCGCTATCGCCA TCGCTATCGCCA TCGCTATC---- --------TCCA TCA CH1 (Type B)
 117 TCGCTATCGCCA TCGCTATCGCCA TCGCTATCGCCA TCGCTATC---- ------------ --------TCCA TCA STD (Reference)
 117 TCGCTATCGCCA TCGCTATCGCCA TCGCTATCGCCA TCGCTATC---- ------------ --------TCCA TCA CH7 (Type C)
 117 TCGCTATCGCCA TCGCTATCGCCA TCGCTATC---- ------------ ------------ --------TCCA TCA R41 (Type D)
 117 TCGCTATCGCCA TCGCTATC---- ------------ ------------ ------------ --------TCCA TCA CH63 (Type E)
 117 TCGCTATCGCCA TC---------- ------------ ------------ ------------ ------------ --A ND7 (Type F)
Figure S2: Tandem repeat variation in housekeeping genes from Plasmodium vivax field isolates.
A) DNA gyrase and B) Ribosomal protein l34a
Tandem repeat unit is underlined. Dash (-) represents deleted nucleotides
