# Supplementary material for: Neutral Polymorphisms in Putative Housekeeping Genes and Tandem Repeats Unravels the Population Genetics and Evolutionary History of Plasmodium vivax in India
Source: PLoS Negl Trop Dis. 2013 Sep 19;7(9):e2425. doi: 10.1371/journal.pntd.0002425 (PMC3777877; doi:10.1371/journal.pntd.0002425)
Supplement: Figure S3 — Expected and observed pairwise differences at seven Plasmodium vivax housekeeping genes. (PPT) [file pntd.0002425.s003.ppt]

## Slide 1
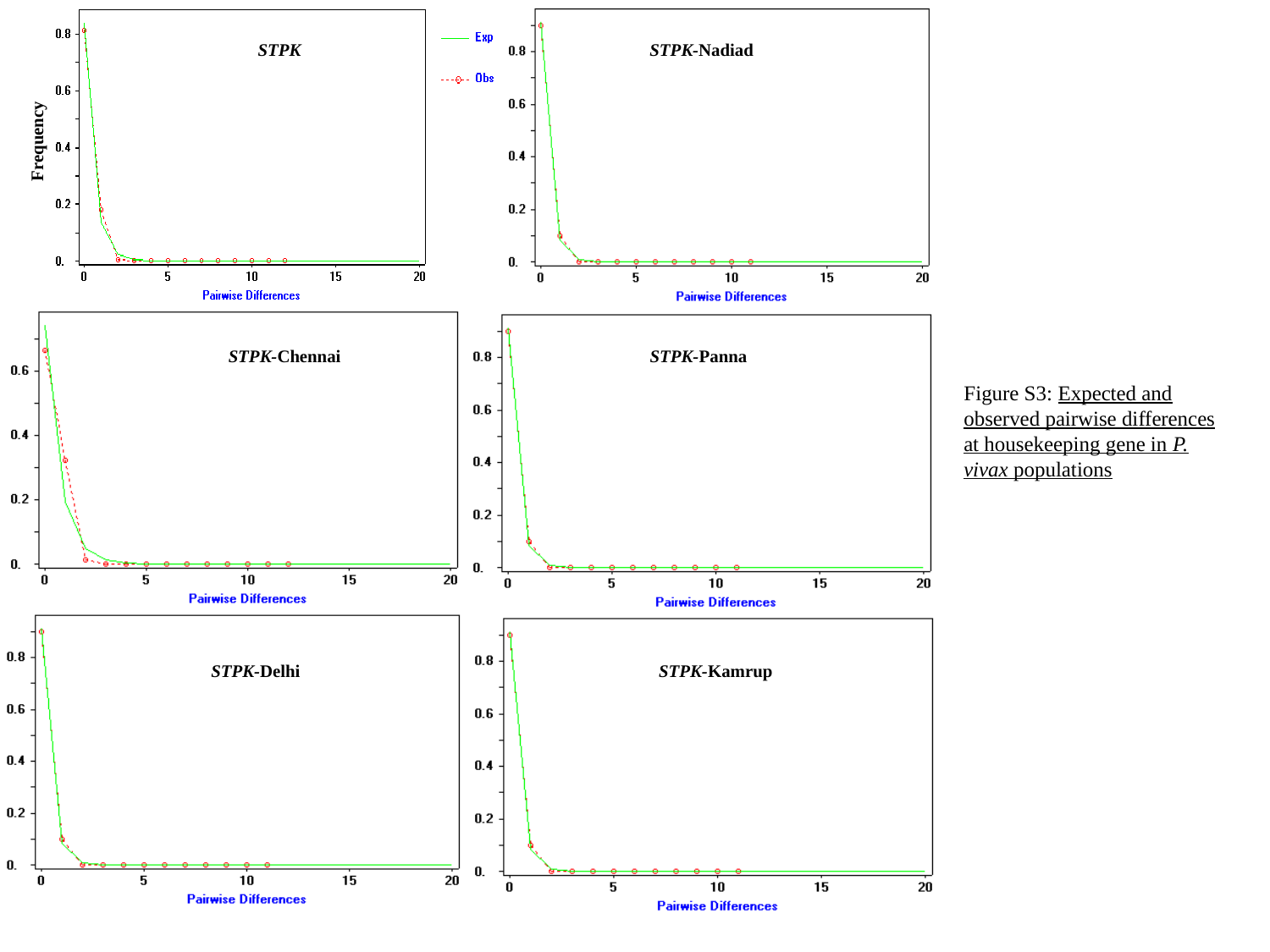

STPK
STPK-Nadiad
Frequency
STPK-Chennai
STPK-Panna
Figure S3: Expected and observed pairwise differences at housekeeping gene in P. vivax populations
STPK-Delhi
STPK-Kamrup
