# Supplementary material for: Neutral Polymorphisms in Putative Housekeeping Genes and Tandem Repeats Unravels the Population Genetics and Evolutionary History of Plasmodium vivax in India
Source: PLoS Negl Trop Dis. 2013 Sep 19;7(9):e2425. doi: 10.1371/journal.pntd.0002425 (PMC3777877; doi:10.1371/journal.pntd.0002425)
Supplement: Table S1 — Primers and amplification conditions. (DOC) [file pntd.0002425.s004.doc]

**Table S1**: Primers and amplification conditions

| **Gene/Locus** | **Primers name** | **Primer sequence (5'-3')** | PCR amplification conditions (temp§/time)  Time (min) Time (second) Time (min) | | | | | Number of cycle | Product size (bp) |
| --- | --- | --- | --- | --- | --- | --- | --- | --- | --- |
| **Housekeeping genes** | | | 1st step | 2nd step | | | 3rd step |  |  |
| *Exonuclease domain* | ED-NF | GCCTGATTTGGTCGATGGCC | 95/4 | 95/30 | 52/30 | 72/40 | 72/5 | 40 | 350 |
|  | ED-NR | GGGTTGGATAGAAGGAATTGCCT |  |  |  |  |  |  |  |
| *Ribosomal protein l35e* | L35e-F | CATGAAGATAGCAACATTACGC | 95/4 |  | 57/30 |  |  | 35 | 600 |
|  | L35e-R | GACCCCCTTTTGCAGAGTAAC |  |  |  |  |  |  |  |
|  | L35e-NF | TGCACGTGAAACGTTACACG |  |  | 50/30 |  |  |  |  |
|  | L35e-NR | GAGTAACGTTAAAGCGTTTC |  |  |  |  |  |  |  |
| *Serine/Threonine protein kinase* | STPK-F | ACTTGATGCAAGGCCTACTC |  |  | 55/30 |  | 72/7 | 40 | 1100 |
|  | STPK-IF# | TTCATTTGCGTGCTGAAGTG |  |  |  |  |  |  |  |
|  | STPK-R | CGTGGCTGCTCGTGTGGAAC |  |  |  |  |  |  |  |
| *Acyle carrier protein (Exon)* | ACPex-F | ACATCGAGAGTCGTGTGC |  |  | 54/30 |  |  |  | 700 |
|  | ACPex-R | CTCCCATGACGAAACCGC |  |  |  |  |  |  |  |
| *Acyle carrier protein (Intron)* | ACPin-F | GAGAATTTACACAAAAGCC |  |  | 50/30 |  |  |  | 730 |
|  | ACPin-R | TTCAGGTCTTTGCGCACTG |  |  |  |  |  |  |  |
| *Adenylate cyclase* | AC-F | ATCTTAGGCTTCTACTCAAAT |  |  |  |  |  | 35 | 600 |
|  | AC-R | GACACACTCATTTGAAGTC |  |  |  |  |  |  |  |
|  | AC-NF | CTACTCAAATAAGGTTAATC |  |  | 52/30 |  |  |  |  |
|  | AC-NR | CCAGATATTGCATTTCCGCG |  |  |  |  |  |  |  |
| *Calcium dependent protein kinase* | CDPK-F | AGGAAGGGGTTCATTCTAAGC |  |  |  | 72/60 |  |  | 1010 |
|  | CDPK-IR# | GATGACTCGGAGATGAAG |  |  |  |  |  |  |  |
|  | CDPK-R | CTTCGAGAGCTGCCGCCT |  |  |  |  |  |  |  |
| *RNA polymerase-II sub unit* | RNAP-F | GTTCCTTCCCTGTTTACC |  |  | 53/30 |  |  | 40 | 980 |
|  | RNAP-IF# | CCAGAAAACATCGACCTG |  |  |  |  |  |  |  |
|  | RNAP-R | TGCAATCGCCCTTCTCCG |  |  |  |  |  |  |  |

| *DNA gyrase* | DG-F | CGCATAGGAGAGGAAGTTC | 95/4 | 95/30 | 52/30 | 72/60 | 72/7 | 40 | 1160 |
| --- | --- | --- | --- | --- | --- | --- | --- | --- | --- |
|  | DG-IF# | GAATGTTGCATTTTGTACTCC |  |  |  |  |  |  |  |
|  | DG-R | AACTCAATTCGCGGAGAGGGA |  |  |  |  |  |  |  |
| *Ribosomal protein l34* | L34-F | GAGGAAGCAACTCACGTC |  |  | 57/30 |  |  | 35 | 1130 |
|  | L34-IF# | GTGCCGACTGCAAAACGG |  |  |  |  |  |  |  |
|  | L34-R | TCGCTCGGCGGTTGTCTG |  |  |  |  |  |  |  |
| *Enolase* | Eno-F | GGCCCACGTAATTACTCGT |  |  | 52/30 |  |  |  | 1200 |
|  | Eno-IR# | GGAAGATACCGACGTTCGT |  |  |  |  |  |  |  |
|  | Eno-R | GCGTATGATACACTTCCGC |  |  |  |  |  |  |  |
| **Minisatellite markers [1]** | | | | | | | | | |
| MiniSat-1 | Mini1-F | ATGCTTCATTGGGTCCAC | 95/4 | 95/30 | 50/30 | 72/40 | 72/7 | 40 | 279 |
|  | Mini1-R | TCGAACAGGACAATGCTG |  |  |  |  |  |  |  |
| MiniSat-2 | Mini2-F | TCACCGGTGGGTCCTTCG |  |  |  |  |  |  | 255 |
|  | Mini2-R | GCAGCGACGAACCGTCAC |  |  |  |  |  |  |  |
| MiniSat-5 | Mini5-F | CAACCTGCAGAGCAATGC |  |  | 55/30 |  |  |  | 254 |
|  | Mini5-R | ACGTTTCTGGGCGACTTC |  |  |  |  |  |  |  |
| MiniSat-6 | Mini6-F | TTGTGCTGTGCTGTGCTG |  |  |  |  |  |  | 172 |
|  | Mini6-R | ACGGTTGGTATGGTCAGG |  |  |  |  |  |  |  |
| MiniSat-8 | Mini8-F | AGCCACAATCCCAACTGC |  |  | 52/30 |  |  |  | 266 |
|  | Mini8-R | TGGTGGTTGTGACTCTAG |  |  |  |  |  |  |  |
| MiniSat-11 | Mini11-F | GGCACAGTGATCATATTCG |  |  | 55/30 |  |  |  | 197 |
|  | Mini11-R | GCGGGTACATAACGCATG |  |  |  |  |  |  |  |
| MiniSat-13 | Mini13-F | GGCACATGAACTTTTCGG |  |  | 52/30 |  |  |  | 258 |
|  | Mini13-R | TTCACCATGGTCCCTTCG |  |  |  |  |  |  |  |
| MiniSat-14 | Mini14-F | CTCTTCGTCGCGTCCAGG |  |  |  |  |  |  | 287 |
|  | Mini14-R | CAGGGTATCCACGACCAG |  |  |  |  |  |  |  |
| MiniSat-16 | Mini16-F | TATGTACTACCTCCACCC |  |  |  |  |  |  | 262 |
|  | Mini16-R | AGCGCGAATATGCATACG |  |  |  |  |  |  |  |
| PvCDPK | CDPK-F | CGCCTCTTTTTCGAGCCC |  |  | 55/30 |  |  |  | 375 |
|  | CDPK-R | CTGCGCCTTCCGCGTCTT |  |  |  |  |  |  |  |

| **Microsatellite markers** | | | | | | | | | |
| --- | --- | --- | --- | --- | --- | --- | --- | --- | --- |
| Msat 38 | Msat38-F | NED AACAACGCAATGTGGATAA | 95/4 | 95/30 | 52/10  47/10 | 72/60 | 72/7 | 35 | 191 |
|  | Msat 38-R | TTACGCTTAACTCATTCGT |  |  |  |  |  |  |  |
| Msat 40 | Msat40-F | 6FAM ATTTGCGTACGGTTAAGAT |  |  |  |  |  |  | 158 |
|  | Msat 40-R | CAGGGTTATTCAATTTGCT |  |  |  |  |  |  |  |
| Msat 50 | MSat50-F | 6FAM CAAAGGAACATGCTCGAT |  |  |  |  |  |  | 128 |
|  | Msat 50-R | GAATTCTGAAGGAATTAGG |  |  |  |  |  |  |  |
| Msat 92 | MSat92-F | 6FAM TCACTGATCTTTTCGCATG |  |  |  |  |  |  | 110 |
|  | Msat 92-R | TAGTAGCATAGTGGTAGTA |  |  |  |  |  |  |  |
| Msat 21 | Msat 21-F | VIC ATCTGCTCAAATCCGAAG |  |  |  |  |  |  | 291 |
|  | Msat 21-R | TATGATGGTAACTTCCGTT |  |  |  |  |  |  |  |
| Msat 73 | Msat 73-F | VIC CACTACATTAGGTGTATAC |  |  |  |  |  |  | 200 |
|  | Msat 73-R | TGATGGTGAAGCTGTTCA |  |  |  |  |  |  |  |
| Msat 128 | Msat128-F | NED TTGAGGAAGTAATACAGGT |  |  |  |  |  |  | 220 |
|  | Msat 128-R | AAGCTCAGCGACTACTTT |  |  |  |  |  |  |  |
| Gomez_1 [2] | N2 F | 6FAM GTAAAAATTCGAAACATC | 95/4 |  | 55/30 | 72/40 |  | 40 | 130 |
|  | N2 R | TTTTATTCACAGTAAAGTGC |  |  |  |  |  |  |  |

§: Temperature (°C), *: time in minute, #: Internal primer.

1. Prajapati SK, Joshi H, Shalini S, Patarroyo MA, Suwanarusk R, et al. (2011) Plasmodium vivax lineages: geographical distribution, tandem repeat polymorphism, and phylogenetic relationship. Malar J 10: 374.

2. Gehde N, Hinrichs C, Montilla I, Charpian S, Lingelbach K, et al. (2009) Protein unfolding is an essential requirement for transport across the parasitophorous vacuolar membrane of Plasmodium falciparum. Mol Microbiol 71: 613-628.
