# Supplementary material for: Neutral Polymorphisms in Putative Housekeeping Genes and Tandem Repeats Unravels the Population Genetics and Evolutionary History of Plasmodium vivax in India
Source: PLoS Negl Trop Dis. 2013 Sep 19;7(9):e2425. doi: 10.1371/journal.pntd.0002425 (PMC3777877; doi:10.1371/journal.pntd.0002425)
Supplement: Table S2 — Characteristic features of Plasmodium vivax mini and microsatellite markers. (DOC) [file pntd.0002425.s005.doc]

Table S2: Characteristic features of *Plasmodium vivax* mini and microsatellite markers.

| **Marker** | **Locus name** | **Chro No.** | **Repeat size** | **Repeat Unit** | **Copy No.** | **Size (bp)** |
| --- | --- | --- | --- | --- | --- | --- |
| **Minisat** | PvCDPK | 4 | 12 | ATTTTGCTTTCC | 25 | 375 |
|  | MiniSat 1 | 2 | 15 | TTTTTCCCCATCTCA | 12.3 | 279 |
|  | MiniSat 2 | 2 | 11 | AACAAAAAAAA | 15.6 | 255 |
|  | MiniSat 5 | 2 | 15 | GGGGAGAGCGGCAAA | 10.1 | 254 |
|  | MiniSat 6 | 10 | 11 | TTTTCTTCCTT | 11.7 | 172 |
|  | MiniSat 8 | 10 | 12 | AACCAGAAATGG | 30.2 | 404 |
|  | MiniSat 11 | 14 | 12 | TGCTGCTCCGAC | 12.8 | 197 |
|  | MiniSat 13 | 14 | 9 | AGGTTAAGC | 11.8 | 258 |
|  | MiniSat 14 | 6 | 12 | GTTGCCGCCGTG | 16 | 287 |
|  | MiniSat 16 | 8 | 12 | ATGTACCTACTG | 16.2 | 262 |
| **Microsat** | MS_38 | 6 | 4 | CATA | 16 | 191 |
|  | MS_40 | 6 | 3 | AAT/C | 13 | 158 |
|  | MS_50 | 6 | 3 | CTA | 19 | 128 |
|  | MS_21 | 2 | 2 | AC | 17.5 | 291 |
|  | MS_73 | 7 | 4 | TCAC/TCAT | 10 | 200 |
|  | MS_92 | 5 | 3 | GAA | 24 | 110 |
|  | MS_128 | 10 | 2 | T/CA | 21 | 220 |
|  | Gomez_1 | UN | 2 | AT | 20 | 130 |

UN: unknown
