# Supplementary material for: Neutral Polymorphisms in Putative Housekeeping Genes and Tandem Repeats Unravels the Population Genetics and Evolutionary History of Plasmodium vivax in India
Source: PLoS Negl Trop Dis. 2013 Sep 19;7(9):e2425. doi: 10.1371/journal.pntd.0002425 (PMC3777877; doi:10.1371/journal.pntd.0002425)
Supplement: Text S1 — Details of study sites. (DOC) [file pntd.0002425.s006.doc]

**Supplementary information:**

**Details of study sites:**

**Delhi** (New Delhi), the capital of India located in the north, is subject to frequent influx of people from all over the country seeking jobs. Malaria in Delhi is transmitted by the mosquito vectors *Anopheles stephensi* and *An. culicifacies* in urban and rural populations, respectively. *Plasmodium vivax* is the predominant malaria parasite species. *P. vivax* transmission occurs in two distinct peaks: a lesser peak in March/April and a major peak in the post-monsoon months of July to October. *P. falciparum* transmission occurs from September/October until November and is interrupted during the winter months (December to January). *P. vivax* malaria in the Delhi region is characterized by a high relapse rate [1,2].

**Nadiad** is a city in a malaria-endemic region in Gujarat state, located in western India. State malaria records reveal that malaria incidence was historically highest in the Kheda district (including Nadiad) in the form of epidemics. Both species of *Plasmodium* (*P. falciparum* and *P. vivax*) are present, though *P. vivax* is predominant (70-80%), and their transmission is mainly during post-monsoon months. Malaria in Nadiad is transmitted by the vectors *An. stephensi* and *An. culicifacies* in urban and rural populations, respectively. Relapses due to vivax malaria have been reported in Nadiad [3,4].

**Panna** is an isolated, hilly, forested area in Madhya Pradesh state (Central India) and is dominated by tribal populations (mainly Gond). Malaria transmission occurs throughout the year with a predominance of *P. falciparum*. *P. vivax* transmission is mainly during March to July. In recent years, the forested area has experienced several malarial epidemics [5,6,7,8,9]. *An. culicifacies* and *An. fluvitalis* are the major malaria vectors in this region.

**Chennai** (Tamil Nadu) is a coastal metropolitan city located in the southern region of the country. Chennai is rife with urban malaria. Large numbers of overhead water tanks in the city provides good conditions for mosquito breeding. Malaria there is mainly (>90%) due to *P. vivax* and is transmitted by *An. stephensi*. Malaria transmission is stable in Chennai and occurs throughout the year. Recently, cases of chloroquine-resistant *P. falciparum* malaria have been reported in Tamil Nadu state [10].

**Kamrup** is in Assam state, located in the northeastern region of the country. *P. falciparum* is the predominant human malaria parasite, though rare cases of *P. vivax* are reported in epidemic form. Northeastern states of India are known to have high prevalence of multi-drug resistant strains [11] and the region is surrounded by international borders. The borders are very porous; illegal migration of people across the borders is common, facilitating import of drug-resistant strains. Malaria transmission vectors in this region are the highly anthropophagic mosquitoes *An. minimus* and *An. dirus.*

**References**

1. Adak T, Sharma VP, Orlov VS (1998) Studies on the Plasmodium vivax relapse pattern in Delhi, India. Am J Trop Med Hyg 59: 175-179.

2. Adak T, Valecha N, Sharma VP (2001) Plasmodium vivax polymorphism in a clinical drug trial. Clin Diagn Lab Immunol 8: 891-894.

3. Sharma RC, Gautam AS, Orlov V, Sharma VP (1990) Relapse pattern of Plasmodium vivax in Kheda district, Gujarat. Indian J Malariol 27: 95-99.

4. Srivastava HC, Sharma SK, Bhatt RM, Sharma VP (1996) Studies on Plasmodium vivax relapse pattern in Kheda district, Gujarat. Indian J Malariol 33: 173-179.

5. Singh N, Chand SK, Mishra AK, Bharti PK, Singh MP, et al. (2006) Epidemiology of malaria in an area of low transmission in central India. Am J Trop Med Hyg 75: 812-816.

6. Singh N, Chand SK, Mishra AK, Nagpal AC (2004) Migration malaria associated with forest economy in central India. Current Science 87: 1696-1699.

7. Singh N, Nagpal AC, Saxena A, Singh MP (2004) Changing scenario of malaria in central India, the replacement of Plasmodium vivax by Plasmodium falciparum (1986-2000). Trop Med Int Health 9: 364-371.

8. Singh N, Sharma VP, Shukla MM, Chand G (1988) Malaria outbreak in Kundam block, district Jabalpur (M.P.). Indian J Malariol 25: 41-49.

9. Singh N, Valecha N, Sharma VP (1997) Malaria diagnosis by field workers using an immunochromatographic test. Trans R Soc Trop Med Hyg 91: 396-397.

10. Eapen A, Ravindran KJ, Joshi H, Dhiman RC, Balavinayagam S, et al. (2007) Detection of in-vivo chloroquine resistance in Plasmodium falciparum from Rameswaram Island, a pilgrim centre in southern India. Ann Trop Med Parasitol 101: 305-313.

11. Sehgal PN, Sharma MID, I. SS, Gogal S (1973) Resistance to chloroquine in falciparum malaria in Assam state, India. J Commun Dis 5: 175-180.
